# Relationship Between Calcium Intake and Impaired Activities of Daily Living in a Japanese Population: NIPPON DATA90

**DOI:** 10.2188/jea.JE20190234

**Published:** 2021-02-05

**Authors:** Mana Kogure, Naho Tsuchiya, Akira Narita, Takumi Hirata, Naoki Nakaya, Tomohiro Nakamura, Atsushi Hozawa, Takehito Hayakawa, Nagako Okuda, Naoko Miyagawa, Aya Kadota, Takayoshi Ohkubo, Yoshitaka Murakami, Kiyomi Sakata, Katsuyuki Miura, Akira Okayama, Tomonori Okamura, Hirotsugu Ueshima

**Affiliations:** 1 Division of Personalized Prevention and Epidemiology, Tohoku Medical Megabank Organization, Tohoku University, Sendai, Japan; 2 Department of Public Health, Hokkaido University Faculty of Medicine, Sapporo, Japan; 3 Department of Health Sciences, Saitama Prefectural University, Saitama, Japan; 4 Research Center for Social Studies of Health and Community, Ritsumeikan University, Kyoto, Japan; 5 Department of Health and Nutrition, University of Human Arts and Sciences, Saitama, Japan; 6 International Center for Nutrition and Information, National Institute of Health and Nutrition, Tokyo, Japan; 7 Department of Public Health, Shiga University of Medical Science, Shiga, Japan; 8 Center for Epidemiologic Research in Asia, Shiga University of Medical Science, Shiga, Japan; 9 Department of Hygiene and Public Health, Teikyo University School of Medicine, Tokyo, Japan; 10 Department of Medical Statistics, Faculty of Medicine, Toho University, Tokyo, Japan; 11 Department of Hygiene and Public Health, Iwate Medical University, Iwate, Japan; 12 Research Institute of Strategy for Prevention, Tokyo, Japan; 13 Department of Preventive Medicine and Public Health, Keio University School of Medicine, Tokyo, Japan

**Keywords:** bootstrap analyses, calcium intake, impaired activities of daily living, nested case-control study, NIPPON DATA90

## Abstract

**Background:**

Major reasons for long-term care insurance certification in Japan are stroke, dementia, and fracture. These diseases are reported to be associated with calcium intake. This study examined the association between calcium intake and impaired activities of daily living (ADL) using the data from NIPPON DATA90, consisting of representative sample of the Japanese population.

**Methods:**

A population-based nested case-control study was performed. A baseline survey was conducted in 1990, followed by ADL surveys of individuals ≥65 years old in 2000. Individuals with impaired ADL and selected age- and sex-matched controls were then identified. We obtained 132 pairs. Calcium intake was energy-adjusted using the residual method. The association between calcium intake and impaired ADL was examined using conditional logistic regression models. To assess the accuracy of the estimates, we conducted bootstrap analyses.

**Results:**

The adjusted odds ratios (ORs) for impaired ADL compared with the group with a calcium intake of <476 mg/day were 0.72 (95% confidence interval [CI], 0.37–1.40) for the 476–606 mg/day group and 0.44 (95% CI, 0.21–0.94) for the ≥607 mg/day group in 2000 (*P* for linear trend = 0.03). After the bootstrap analyses, the inverse relationship unchanged (median OR per 100-mg rise in calcium intake, 0.87 [1,000 resamplings]; 95% CI, 0.76–0.97).

**Conclusions:**

After bootstrap analyses, calcium intake was inversely associated with impaired ADL 10 years after the baseline survey.

## INTRODUCTION

Japan is known to have the longest life expectancy and healthy life expectancy (HALE) in the world.^1^ The life expectancy in 2013 was 80.05 years for Japanese men and 86.39 years for Japanese women, compared with 76.04 years and 81.96 years, respectively, in 1990. On the other hand, the HALE in 2013 was 71.11 years for men and 75.56 years for women, compared with 68.09 years and 72.24 years, respectively, in 1990.^1^ Thus, both the life expectancy and HALE have increased over the last two decades. However, the gap between life expectancy and HALE has increased somewhat. The gap in 2013 was 8.94 years for men and 10.83 years for women, compared with 7.95 years and 9.72 years, respectively, in 1990. Reducing the gap between life expectancy and HALE by increasing the HALE is an important objective. One means of increasing the HALE is to prevent reductions in activities of daily living (ADL). The Global Burden of Disease 2013 also discussed the gap between life expectancy and HALE.^1^ They recommended the use of country-specific assessments of disability-adjusted life-years (DALYs) and HALE when forming appropriate health policies.

In Japan, the Comprehensive Survey of Living Conditions has been used to investigate the reasons for long-term care insurance (LTCI) certification, which is required for frail older persons to receive caregiving services.^2^ The 2013 survey reported that the major reasons for LTCI certification were stroke, dementia, infirmity due to age, and fracture.^3^ Among these reasons, stroke, dementia, and bone loss are reported to be associated with calcium intake.^4^^–^^8^ These studies suggested that calcium intake might prevent impairments in ADL, but only one cohort study has investigated the impact of calcium intake on impaired ADL.^9^ The present study examined the association between calcium intake and impaired ADL in a Japanese population using NIPPON DATA90, consisting of subjects who participated in the National Nutrition Survey in Japan (NNSJ).

## METHODS

### NIPPON DATA90

The National Integrates Project for Prospective Observation of Non-communicable Disease And its Trends in Aged, 1990 (NIPPON DATA90) was a prospective cohort study examining a total of 10,956 community-based men and women, 30 years and older in age, from 300 randomly selected areas throughout Japan.^10^^–^^12^ The baseline survey performed in 1990 consisted of a physical examination, blood tests, and self-administered questionnaires on medical history and lifestyle. A total of 8,383 community residents (men, 3,503; women, 4,880; 30 years and older) from 300 randomly selected areas participated in the survey, corresponding to a participation rate of 76.5% (8,383 of 10,956). Participants were followed until November 15, 2010. In addition, NIPPON DATA90 also conducted a survey of ADL for subjects aged 65 years and older in 2000. The ADL survey was conducted through face-to-face interviews at home, telephone interviews, and a questionnaire sent via mail or other methods. The Institutional Review Board of Shiga University of Medical Science (No. 12-18) approved this study.

### Nutrition survey

Data on calcium intake was obtained from the NNSJ in 1990. The NNSJ has been conducted every year since 1948. Until 1994, nutrient intakes were calculated for each household. We estimated the individual nutrient intakes in 1990 by dividing the household nutrient intake in 1990 proportionally using the average intakes for sex and age groups calculated for the NNSJ in 1995. Details of the methods used to perform the nutrition survey and to estimate the individual nutrient intakes have been described elsewhere.^13^

### Matching

Cases were defined as participants with impaired ADL in 2000. Candidates for control matching (same sex and ±5 years in age) were selected for each case. The controls were then randomly selected from among the candidates (case:control = 1:1).

### Study participants

**Figure 1.**
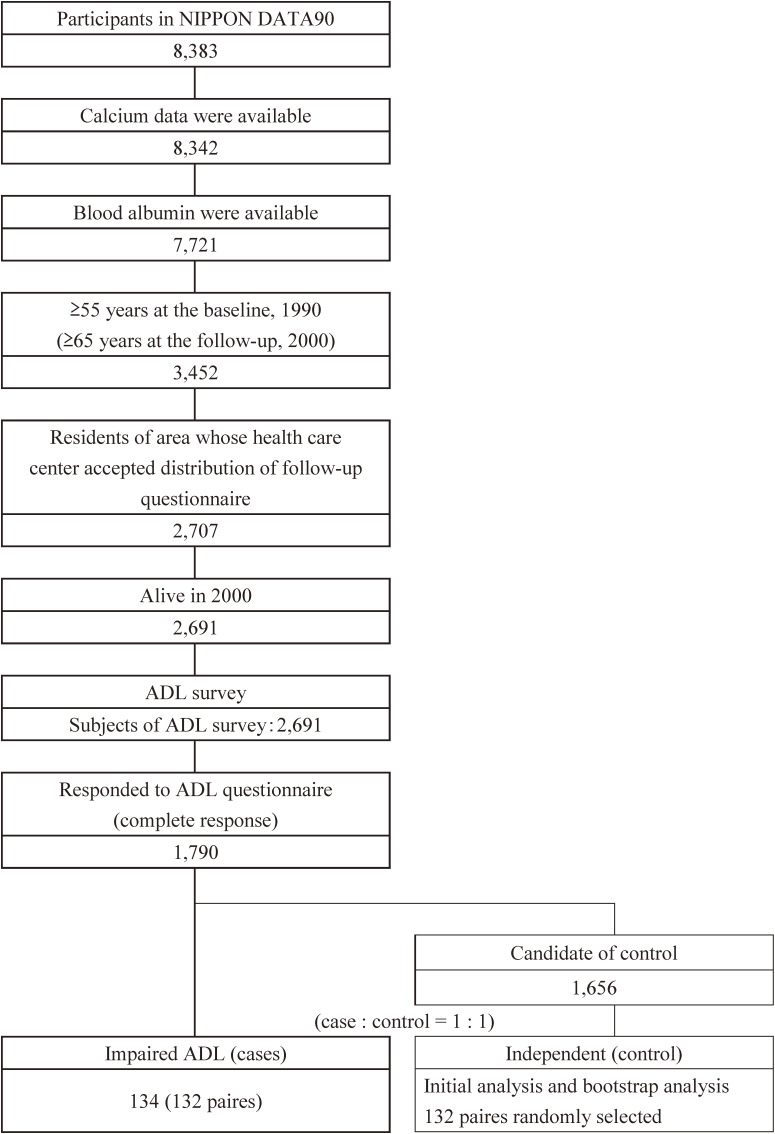
Flow chart of study participants. Participants who met the following criteria were included in the analyses: 1) Subjects who were 55 years old or more at baseline, 2) availability of calcium data, 3) availability of blood albumin data, 4) resident of area whose health care center accepted distribution of follow-up questionnaire, 5) alive at each time point, and 6) responded to ADL questionnaire. ADL, activities of daily living; NIPPON DATA90, The National Integrates Project for Prospective Observation of Non-communicable Disease And its Trends in Aged, 1990.

The present study was a nested case-control study using data from the NIPPON DATA90. In the main analysis, we examined the relationship between calcium intake in 1990 and impaired ADL in 2000. Participants who met the following criteria were included in the analyses: 1) Subjects who were 55 years or older at baseline, 2) availability of calcium data, 3) availability of blood albumin data, 4) resident of area whose health care center accepted distribution of follow-up questionnaire, 5) alive at each time point, and 6) responded to ADL questionnaire (Figure 1). Overall, 1,790 participants (cases: 134, controls: 1,656) were included as candidates for matching. After age-sex matching, 132 pairs were matched for the analyses of data from 2000.

### Evaluation of ADL

Participants were asked about five basic ADL items (feeding, dressing, bathing, toileting and transfer: walking indoors), modified from the report by Katz et al.^14^ Impaired ADL was defined as the need for partial or full support for any of these five basic ADL items.

### Statistical analysis

To compare the baseline characteristics between the participants with impaired ADL and those with normal ADL, we used conditional logistic regression models for continuous variables (age, albumin, systolic blood pressure [SBP], diastolic blood pressure [DBP], and energy intake) and categorical variables (sex, body mass index [BMI], sports, smoking status, and drinking status). To evaluate the dose-response relationships between calcium intake and the above-mentioned variables, we used a general linear model for the continuous variables and a logistic regression model for the categorical variables. Calcium intake was energy-adjusted using the residual method^15^ and was classified into tertiles.

Conditional logistic regression models were applied to examine the relationship between calcium intake and impaired ADL. We included potential confounding factors such as age (years, continuous), energy intake (kcal, continuous), serum albumin (g/dL, continuous), BMI (<18.5 kg/m^2^, 18.5–24.9 kg/m^2^ and ≥25.0 kg/m^2^), sports (yes, “I have exercise”; no, “I cannot perform exercise for health reasons” or “I cannot perform exercise without any health reasons”), smoking status (current, ex, almost never), drinking status (current, ex, almost never) and hypertension (SBP <140 mm Hg and DBP <90 mm Hg and no use of antihypertensive drugs, SBP ≥160 mm Hg or DBP ≥100 mm Hg or use of antihypertensive drugs, other intermediate group). *P* values for linear trends were calculated using the categories of calcium intake.

Because the results of the conditional logistic regression analysis depend on the selection of the controls, replicated bootstrap sampling^16^ of the controls were performed to calculate the 95% confidence interval (CI) for the odds ratio (OR) per 100-mg rise in calcium intake. The number of samplings was set at 1,000 times to assess the accuracy of the estimates obtained from the subsets.

*P* values of <0.05 were considered statistically significant. All the analyses were performed using SAS version 9.4 for Windows (SAS Inc., Cary, NC, USA).

## RESULTS

Table 1 shows the baseline characteristics of participants according to case and control. One hundred and thirty-two pairs were matched for the analyses. Overall, the mean age was 69.5 (standard deviation, 7.0) years. Of the 264 participants, 146 (55.3%) were women. There were no differences in age and sex between the cases and controls. Table 1 shows the characteristics of the participants according to calcium intake. The proportion of men decreased as the calcium intake increased (*P* for linear trend <0.01). The proportion of current drinkers also decreased as the calcium intake increased (*P* for linear trend = 0.01).

**Table 1.** Baseline characteristics of study participants in 2000, NIPPON DATA90 (*n* = 264; 132 pairs)

	Cases	Controls	*P* value	Calcium intake (energy-adjusted, mg/day)			*P* for linear trend^a^
				1^st^ tertile	2^nd^ tertile	3^rd^ tertile	
				(<476 mg/day)	(476–606 mg/day)	(≥607 mg/day)	
Number of participants	132	132		89	88	87	
Age, years, mean (SD)	69.7 (6.9)	69.4 (7.2)	<0.01	68.9 (6.5)	70.3 (6.8)	69.3 (7.8)	0.69
Sex (%)							<0.01
Men	59 (44.7)	59 (44.7)	—	55 (61.8)	29 (33.0)	34 (39.1)	
Women	73 (55.3)	73 (55.3)	—	34 (38.2)	59 (67.1)	53 (60.9)	
BMI, kg/m^2^, (%)							
<18.5 kg/m^2^	15 (11.4)	12 (9.1)	0.56	12 (13.5)	11 (12.5)	4 (4.6)	0.06
18.5–24.9 kg/m^2^	82 (62.1)	91 (68.9)	0.23	55 (61.8)	56 (63.6)	62 (71.3)	0.19
≥25.0 kg/m^2^	35 (26.5)	29 (22.0)	0.36	22 (24.7)	21 (23.9)	21 (24.1)	0.93
Systolic blood pressure, mm Hg, mean (SD)	146.8 (22.2)	146.0 (20.2)	0.74	147.1 (23.6)	145.8 (20.1)	146.3 (19.9)	0.81
Diastolic blood pressure, mm Hg, mean (SD)	83.0 (13.7)	81.7 (12.2)	0.40	82.9 (12.1)	80.9 (13.2)	83.3 (13.7)	0.87
Sports (%)							0.79
Yes	35 (26.5)	33 (25.0)	0.77	23 (25.8)	21 (23.9)	24 (27.6)	
No	97 (73.5)	99 (75.0)		66 (74.2)	67 (76.1)	63 (72.4)	
Smoking status (%)							
Current-smoker	33 (25.0)	31 (23.5)	0.76	30 (33.7)	19 (21.6)	15 (17.2)	0.01
Ex-smoker	24 (18.2)	23 (17.4)	0.86	19 (21.4)	9 (10.2)	19 (21.8)	0.94
Never-smoker	75 (56.8)	78 (59.1)	0.59	40 (44.9)	60 (68.2)	53 (60.9)	0.03
Drinking status (%)							
Current-drinker	31 (23.5)	34 (25.8)	0.56	31 (34.8)	18 (20.5)	16 (18.4)	0.01
Ex-drinker	11 (8.3)	2 (1.5)	0.03	4 (4.5)	3 (3.4)	6 (6.9)	0.47
Never-drinker	90 (68.2)	96 (72.7)	0.29	54 (60.7)	67 (76.1)	65 (74.7)	0.04
Albumin, g/dL, mean (SD)	4.3 (0.3)	4.3 (0.2)	0.70	4.3 (0.3)	4.3 (0.3)	4.3 (0.3)	0.33
Energy intake, kcal/day, mean (SD)	1,888 (435)	1,935 (525)	0.36	1,883 (467)	1,833 (490)	2,020 (473)	0.06
Calcium intake, mg/day, mean (SD)	551 (183)	584 (182)	0.14	400 (59)	538 (38)	770 (162)	—
Calcium intake, mg/day, median (IQR)	549 (452–697)	520 (439–636)		405 (369–447)	537 (506–566)	729 (671–794)	

BMI, body mass index; IQR, inter quartile range; SD, standard deviation.

^a^A conditional logistic regression model was used for continuous variables and categorical variables. The general linear model for continuous variables and logistic regression model for categorical variables.

Table 2 shows the association between calcium intake and impaired ADL in 2000 in a randomly sampled subset. The adjusted model without drinking status is shown (*P* for linear trend = 0.13). Finally, the fully adjusted odds ratio (aOR) compared with the group with a calcium intake of <476 mg/day was 0.72 (95% CI, 0.37–1.40) for the 476–606 mg/day group and 0.44 (95% CI, 0.21–0.94) for the ≥607 mg/day group. The risk of impaired ADL decreased with calcium intake in a dose-dependent manner (*P* for linear trend = 0.03).

**Table 2.** Relation between calcium intake and impaired activities of daily living (ADL) in 2000 (*n* = 264; 132 pairs)

	Baseline calcium intake (mg/day)^a^			

	1^st^ tertile (<476 mg/day)	2^nd^ tertile (476–606 mg/day)	3^rd^ tertile (≥607 mg/day)	*P* for linear trend
Number of impaired ADL/Number of subjects	48/89	47/88	37/87	
Crude OR (95% CI)	1.00 (Reference)	0.99 (0.56–1.73)	0.60 (0.32–1.12)	
Model1 OR (95% CI)^b^	1.00 (Reference)	0.93 (0.50–1.71)	0.58 (0.29–1.14)	0.13
Model2 OR (95% CI)^c^ (Fully adjusted model)	1.00 (Reference)	0.72 (0.37–1.40)	0.44 (0.21–0.94)	0.03

BMI, body mass index; CI, confidence interval; DBP, diastolic blood pressure; OR, odds ratio; SBP, systolic blood pressure.

^a^Calcium intake (mg/day) was energy-adjusted using the residual method.

^b^Model 1 OR (95% CI) were calculated using conditional logistic regression models and were adjusted for age (continuous), energy intake (kcal, continuous), serum albumin (g/dL, continuous), BMI (<18.5 kg/m^2^, 18.5–24.9 kg/m^2^, ≥25.0 kg/m^2^), sports (yes, no), smoking status (current, ex, never), hypertension (SBP <140 mm Hg and DBP <90 mm Hg and no use of antihypertensive drugs, SBP ≥160 mm Hg or DBP ≥100 mm Hg or use of antihypertensive drugs, other intermediate group).

^c^Model 2 OR (95% CI) (Fully adjusted model): model 1 + drinking status (current, ex, never).

We also calculated the bootstrap-corrected estimates of the OR per 100 mg in calcium intake for impaired ADL in 2000. The OR for impaired ADL in 2000 had a median of 0.87 and a 95% CI of 0.76–0.97.

## DISCUSSION

We examined the relationship between calcium intake and impaired ADL in a Japanese population using NIPPON DATA 90. Calcium intake was inversely associated with the risk of impaired ADL in 2000, 10 years after the baseline survey. Especially the risk of impaired ADL was significantly lower in the group within the third tertile of calcium intake. After bootstrap analyses, an inverse relationship was still observed between calcium intake and impaired ADL.

In previous Japanese studies, the incidence or mortality of stroke was significantly lower for individuals in a higher dietary calcium intake group, compared with those in a lower dietary calcium intake group.^5^^,^^6^ Additionally, a meta-analysis of prospective cohort studies showed that calcium intake was inversely associated with the risk of stroke in Asian populations.^4^ Ozawa et al reported that dietary calcium intake was also inversely associated with the risk of dementia.^7^ Nakamura et al reported that a low-dose calcium supplement can effectively slow bone loss in the lumbar spine.^8^ We considered that calcium intake might be associated with impaired ADL via diseases resulting in impaired ADL, such as stroke, bone loss, and dementia. Accordingly, the risk of impaired ADL may decrease with calcium intake. As adjustment of drinking status made the result statistically significant, further analysis might be required to clarify this issue.

However, one prospective cohort study in France has shown conflicting results. Vercambre et al reported that the association between calcium intake and instrumental ADL was not statistically significant among French women.^9^ Regional differences in average calcium intake may explain the discrepancy between our results and their results. In fact, the National Health and Nutrition Survey of Japan reported that the mean daily calcium intake was about 500 mg, which is lower than the current recommended amount (650–800 mg/day) for adults.^17^^,^^18^ In Japan, calcium intakes are lower than in other countries, such as the United States or France.^19^^,^^20^ The average calcium intake for subjects aged 1 year and older ranges from 918 to 1,296 mg/day in the United States.^19^ Similarly, the average calcium intake for subjects aged 18 to 79 years was 930 mg/day in France.^20^ Because the effect of calcium on the prevention of stroke or bone loss might be larger when calcium intake is insufficient, the beneficial effect of increasing the calcium intake on ADL may be limited to areas with low calcium intake. Although calcium intake is lower in Japan than in other countries, the risk of impaired ADL was significantly lower in the group within the third tertile of calcium intake. Therefore, it was suggested that the recommended amount of calcium intake at that time was appropriate.

To our knowledge, this is the first report to discuss calcium intake and impaired ADL in a country with a low calcium intake. We believe that these findings might be applicable to other areas with low calcium intakes.

The present study had several strengths. First, we confirmed the accuracy of the significant association between calcium intake and impaired ADL using bootstrap analyses. The bootstrap resampling technique enabled us to minimize the sampling bias. Second, we performed age-sex matched analyses to avoid confounding from age and sex, which are very strong predictors of ADL. To our knowledge, this is the first report to examine calcium intake and impaired ADL using bootstrap analyses. Third, because the NNSJ evaluated nutrient intake using the food weighing method, its estimated accuracy is considered to be higher than that of the food frequency questionnaire generally used in epidemiological studies.

Our study also had some limitations. First, we did not obtain information about calcium supplementation and the intake of drugs, such as diuretics, steroids, or vitamin D, that can influence calcium dynamics. If supplement users had been included in the analyses, the effect of calcium intake on impaired ADL would probably have been underestimated. Second, information on the specific causes of impaired ADL was not available. If this kind of information had been obtained, we would have been able to clarify the association between calcium intake and impaired ADL according to specific causes of impairment. Third, the follow-up rate was not very high (66.5%). If we had chosen a typical prospective cohort study design, our observed results would have been biased. However, we believed that the nested case-control study design enabled us to avoid such bias. Fourth, we could not confirm the presence or absence of impaired ADL at the baseline survey. However, because the NIPPON DATA90 participants all underwent health examination on foot, we considered that they might be independent in ADL. Fifth, in this analysis, calcium intake was classified into tertiles in the group of combined cases and controls. Therefore, the quantile of the population distribution might not be representative. However, in view of clarifying the dose-response relationship between calcium intake and impaired ADL, we considered our approach to be appropriate. Finally, we used the individual calcium intake based on the proportion of individual’s intake for the household’s intake evaluated via the weighing methods. Since the weighing was not based on the directly measured proportion, we considered that we should raise this point as a limitation.

In conclusion, in a nested case-control study, the risk of impaired ADL decreased with calcium intake in 2000, 10 years after the baseline survey. This inverse relationship was confirmed using bootstrap analyses. Increasing the dietary calcium intake might prevent impaired ADL, thereby extending the HALE in nations with low calcium intakes.
